# A Model-Based Method for Gene Dependency Measurement

**DOI:** 10.1371/journal.pone.0040918

**Published:** 2012-07-19

**Authors:** Qing Zhang, Xiaodan Fan, Yejun Wang, Mingan Sun, Samuel S. M. Sun, Dianjing Guo

**Affiliations:** 1 School of Life Sciences and the State Key Laboratory of Agrobiotechnology, The Chinese University of Hong Kong, Shatin, Hong Kong SAR, China; 2 Department of Statistics, The Chinese University of Hong Kong, Shatin, Hong Kong SAR, China; Hebrew University at Jerusalem, The Alexander Silberman Institute of Life Sciences, Israel

## Abstract

Many computational methods have been widely used to identify transcription regulatory interactions based on gene expression profiles. The selection of dependency measure is very important for successful regulatory network inference. In this paper, we develop a new method–DBoMM (Difference in BIC of Mixture Models)–for estimating dependency of gene by fitting the gene expression profiles into mixture Gaussian models. We show that DBoMM out-performs 4 other existing methods, including Kendall’s tau correlation (TAU), Pearson Correlation (COR), Euclidean distance (EUC) and Mutual information (MI) using *Escherichia coli, Saccharomyces cerevisiae, Drosophila melanogaster*, *Arabidopsis thaliana* data and synthetic data. DBoMM can also identify condition-dependent regulatory interactions and is robust to noisy data. Of the 741 *Escherichia coli* regulatory interactions inferred by DBoMM at a 60% true positive rate, 65 are previously known interactions and 676 are novel predictions. To validate the new prediction, the promoter sequences of target genes regulated by the same transcription factors were analyzed and significant motifs were identified.

## Introduction

DNA microarray technology has become a vital tool for global transcriptome analysis and complex gene regulatory network (GRN). An ample amount of computational methods, such as co-expression network [1]–[2], Boolean network [5], [6], differential equation [7], [8], information theory [9], [10], relevance network [11] and Bayesian network (BN) [12]–[14], have been widely adopted to infer the GRN using microarray data.

A fundamental step in gene regulatory network inference is to identify pair-wise dependency, or more specifically, to determine whether a gene directly controls the expression of another [15]. The selection of dependency measure is probably more important than the selection of optimization algorithm [4], [16] for successful identification of gene interactions and therefore the whole regulatory networks. When measuring gene dependency, the expression profiles are treated as vectors in certain space and the pair wise distances are computed [16]. This strategy is used by Pearson correlation (COR), Euclidean distance (EUC), Manhattan metric (MAN), Cosine correlation (EISEN), Spearman correlation (SPEAR), Kendall’s 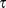 correlation (TAU) [17], etc. Alternatively, the natural pairing of observations is ignored, and the gene expression profiles are assumed to be sampled from different probability distributions. The dependency between two genes is therefore represented by the difference between two distributions. Such strategy is adopted in Kullback-Leibler information (KLI) 18,19 and Mutual information (MI) [20].

COR, EUC and TAU have been widely used as dependency measure by quantifying the similarity or distance of gene expression profiles [21]–[30]. However, these three methods bear obvious limitations. For example, COR is based on the assumption that gene expression profiles are linearly related and it is unable to differ interactions from indirect interactions. The partial correlation, as a modified version of COR by conditioning on all other genes, can measure direct regulatory interactions [31], but it is also limited to linear relationship. Moreover, both COR and EUC are sensitive to noise and outliers [32] and require complete gene expression profiles as input. This has hindered their wide application because microarray data often contain missing gene expression values.

In contrast, mutual information (MI), a well known method in information theory [20], measures the dependency of distributions. In theory, MI can detect any dependence between distributions [33], [34], and it has been widely used to analyze gene expression data [4], [10], [26], [34], [35]. MI is also robust to noise, outliers and missing data. However, the calculation of MI requires the discretization of continuous gene expression values and most discretization methods used rather arbitrary histogram based procedure [10], [34], [36].

In this paper, we describe a method of gene dependency measurement based on the model probability difference between joint modeling and independent modeling of the given data. Specifically, the difference in Bayesian Information Criterion (BIC) between the joint and the marginal distribution models of two genes is used to measure the gene dependency. We assume that joint and the marginal distributions follow a bivariate and two univariate mixture Gaussian distributions respectively. Because this method is based on distributions estimation, it is relatively insensitive to noise, outliers and missing data. In addition, it does not restrict that interacting genes are linearly related. The clustering ability of the mixture model can reflect the condition-dependent relationships between genes [37], [38]. The statistical parameters inferred from gene expression profile can also be used to predict the dynamics of functionally related genes. The efficacy of the proposed model was validated using *Escherichia coli (E.coli), Saccharomyces cerevisiae (Yeast), Drosophila melanogaster (Drosophila), Arabidopsis thaliana (Arabidopsis)* and synthetic datasets.

## Results

### A Comparison with EUC, MI, and COR, TAU

The regulatory networks from RegulonDB [39] and YEASTRACT [40]–[42] are used as reference networks. The interactions between all the transcription factors (TFs) and all the target genes in the reference networks are defined as the background interactions (excluding those real interactions). To determine whether the 5 methods (DBoMM, MI, TAU, COR and EUC) can discriminate the real and the background interactions, the two-sample t-test is used to test whether the scores from real interactions have a mean value bigger (DBoMM, MI and COR) or smaller (EUC and TAU) than that of background interactions.

Table 1 provides the mean scores, standard deviations and the p-values of the t-test. For *E.coli* and synthetic datasets, DBoMM, MI and TAU can distinguish the real interactions from the background but COR cannot (Table 1). EUC works only on synthetic data. For *Yeast* dataset, though the p-values from COR and TAU are smaller than 0.05, the means of scores from real and background interactions are very close. Overall, none of the methods can distinguish the real interactions from the background based on *Yeast* dataset. Previous research [15] also suggested that due to more complex regulatory networks in eukaryotes, other information should be integrated for more accurate prediction of regulatory interactions.

**Table 1:** The distributions of different similarity scores.

	*E.coli*					*Yeast*					Synthetic				
	Real		Background		P.value	Real		Background		P.value	Real		Background		P.value
	mean	sd	mean	sd		mean	sd	mean	sd		mean	sd	mean	sd	
*DBoMM*	138.80	148.88	91.95	89.74	2.12e-79	−3.74	9.26	−3.50	8.47	1	363.26	427.58	16.52	207.88	3.73e-261
MI	0.26	0.14	0.20	0.09	4.69e-114	0.39	0.10	0.40	0.09	1	0.42	0.39	0.11	0.19	2.16e-265
COR	0.69	0.22	0.76	0.17	1	0.17	0.13	0.17	0.12	0.0002	0.44	0.28	0.81	0.25	1
EUC	42.40	24.12	38.18	24.38	1	4.81	1.52	4.56	1.37	1	6.45	3.32	8.02	2.67	1.87e-105
TAU	0.78	0.16	0.82	0.13	1.86e-44	0.88	0.09	0.89	0.09	0.01	0.57	0.26	0.87	0.18	0

**Figure 1:**
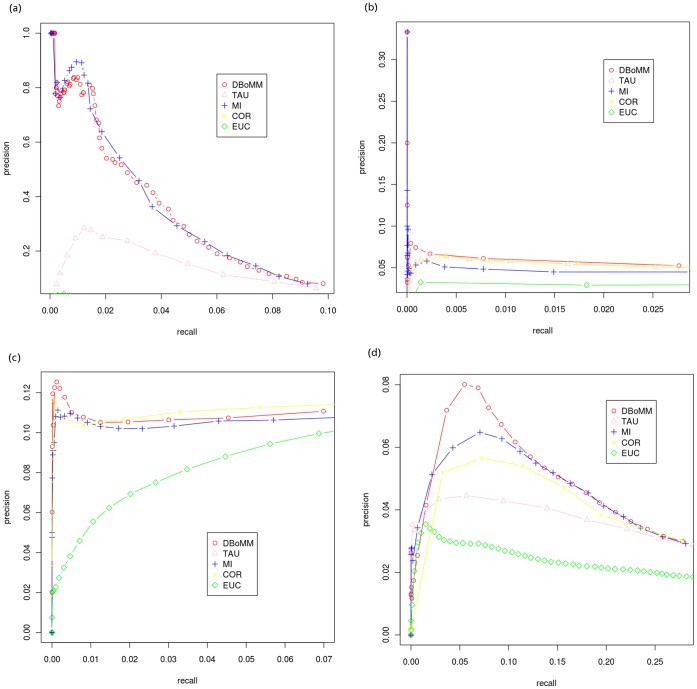
A comparison of different methods using PR-curve. (a). *E.coli* dataset and the reference network from RegulonDB; (b). *Yeast* datset and the reference network from YEASTRACT; (c). *Drosophila* dataset and the reference network from DroID; (d). *Arabidopsis* datset and the reference network from AGRIS. X axis: recall; Y axis: precision. In general, DBoMM out-performs other 4 methods using various datasets.

We then quantitatively compared the performance of the 5 methods using Precision-Recall curve (PR-curve) and the results are shown in Figure 1. The performance of DBoMM is comparable to that of MI when *E.coli* data was used, and both methods are much more effective compared to EUC, COR and TAU. DBoMM out-performs the other 4 methods when *Yeast* and *Arabidopsis* data are used. DBoMM and COR perform similarly using *Drosophila* dataset, and both are better than MI, EUC and TAU. DBoMM performs the best when synthetic dataset is used (Figure S1). In general, DBoMM gives the best performance among these 5 methods.

### Significant Motif is Identified in the Promoters of Predicted Genes

DBoMM is adopted to infer an *E.coli* regulatory network (Figure S2) consisting of 468 genes and 741 regulatory interactions at 60% precision (Figure 1a). Among the 741 interactions, 65 can be validated by RegulonDB. Using MI, a regulatory network with 407 genes and 618 regulatory interactions was inferred. Of the 618 regulatory interactions, 66 can be validated by RegulonDB. Among all the predicted interactions, 424 were inferred by both DBoMM and MI, accounting 57% and 68% of the total interactions respectively. We only extracted the interactions between the 328 known or predicted transcription factors (TFs) and the 4,345 genes to enable clear biological interpretation, assignment of direction (from transcription factors to non–transcription factor genes), and validation of the predictions.

Sequence analysis was conducted to detect the possible TF binding motifs in the promoter regions of the predicted target genes. TFs predicted to regulate 5 or more operons with at least 60% confidence were selected (28 in total). Of these 28 TFs, the binding motifs are known for *FliA, LexA, Fnr, DnaA, Nac* and *PurR* (http://prodoric.tu-bs.de/) [43]. MEME multiple alignment program [44] was used to analyze the upstream sequence (−1 to −150 bp) of the predicted target genes and 4 known motifs were detected (*FliA, LexA, DnaA and Nac* binding motif).

**Figure 2:**
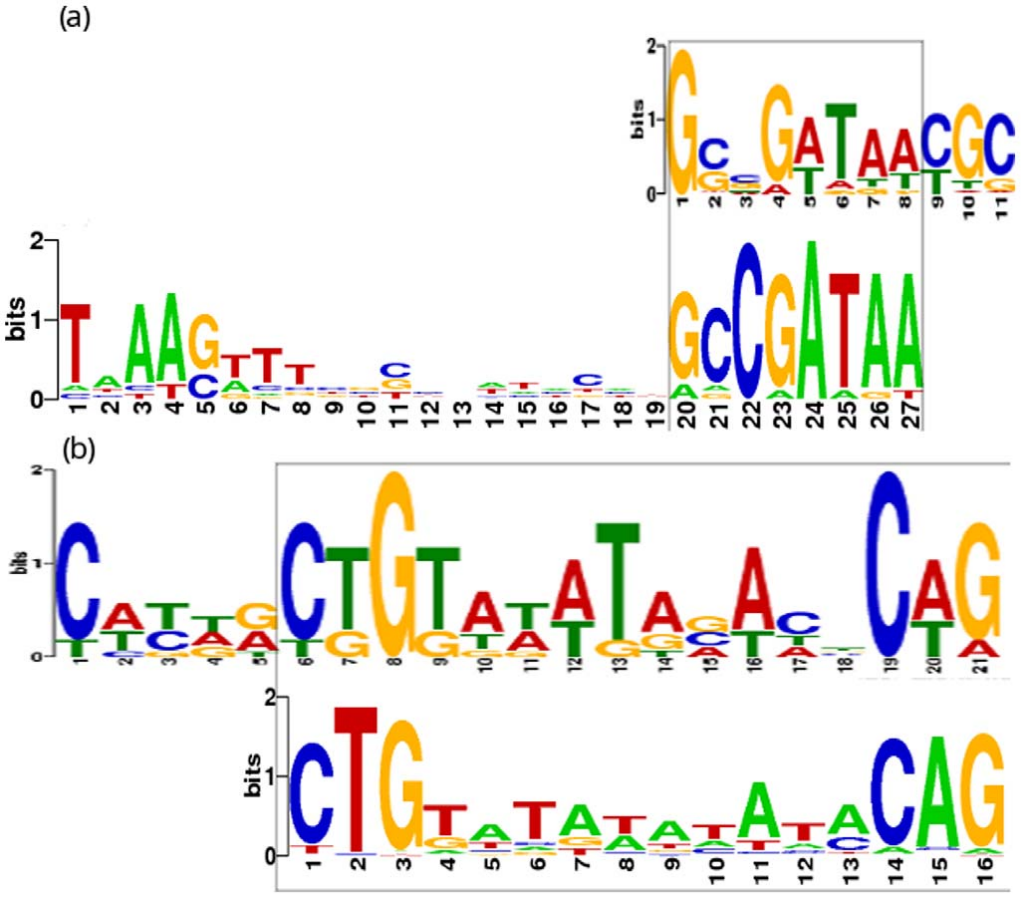
Motifs detected for TF 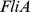 and 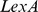. (a). The 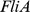 regulatory motif detected in the promoters of the 19 inferred target operons(upper) compared to the motif identified in PRODORIC. (b). The 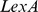 regulatory motif detected in the promoters of 8 inferred target operons(upper) compared to the motif identified in PRODORIC(lower).

*FliA* is a minor sigma factor activating the transcription initiation of a number of genes involved in motility. Notably, most of the target genes are required for flagella synthesis. From DBoMM prediction, *FliA* regulates 52 genes that can be organized into 19 operons. And 40 out of the 52 genes can be validated by RegulonDB. Interestingly, all the operon promoters of the 19 genes contain a significant motif almost identical to the known canonical *FliA* motif (Figure 2a).

*LexA* represses the transcription of several genes involved in cellular response to DNA damage or inhibition of DNA replication [45], [46] as well as its own synthesis [47]. From the predicted regulatory network, *LexA* regulates 10 genes that can be organized into 9 operons. The identical *LexA* regulatory motif can be found in 8 out of the 9 operon promoters (Figure 2b), and 4 of the them can be validated by RegulonDB. The motif information for other 2 TFs can be found in Figure S3.

### DBoMM is Robust Against Noise

**Figure 3:**
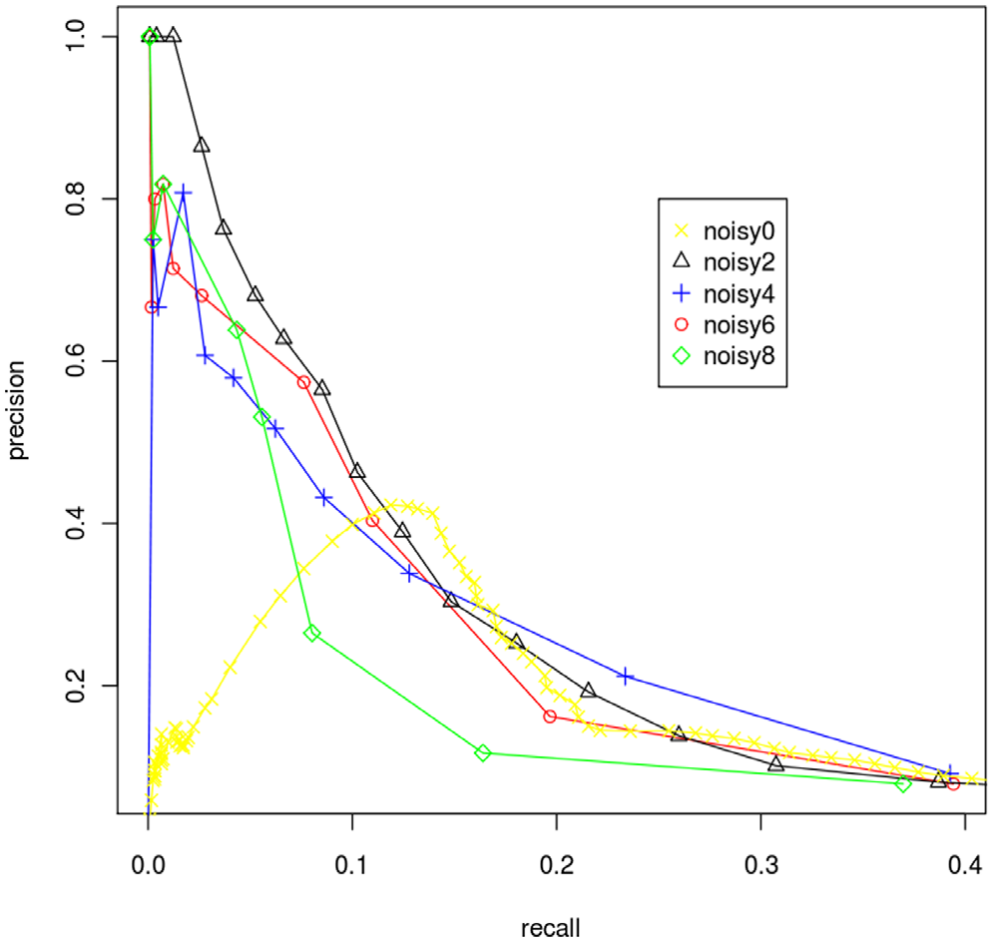
DBoMM is robust to noise. Different levels of noise are introduced to the datasets. The numbers in the legend correspond to the noise levels, e.g. “noisy2” means 20% of noise introduced. DBoMM remains stable with up to 60% of noise. X axis: recall; y axis: precision.

A good estimator should be robust against noise. To test the robustness of DBoMM, we used SynTReN [48], an artificial synthetic dataset generator, to generate simulated gene expression profiles with various noise levels. We then plotted the PR-curves using simulated datasets (Figure 3). Similar performance was achieved when 20%,40% and 60% of noise level was introduced. The precision decreased greatly at 80% of noise level. We also tested the same dataset with MI, COR, EUC and TAU, and the result showed that only MI perform similar to DBoMM, whereas the other 3 methods are not robust (Figure S4). This is because DBoMM and MI are based on the probability distribution, which is more robust to noise.

### DBoMM is Able to Identify Condition-dependent Regulatory Interaction

**Figure 4:**
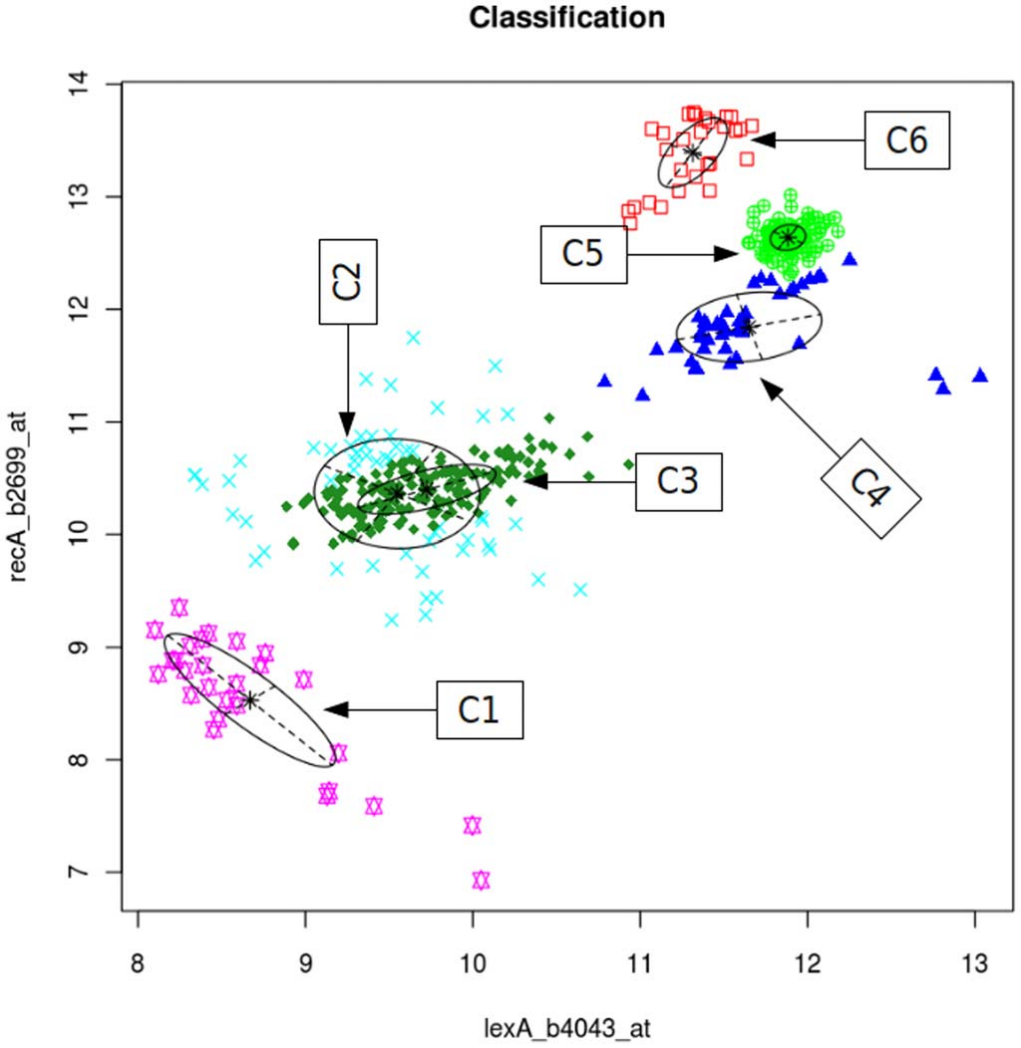
DBoMM can identify the conditional dependent regulatory interactions between two genes. The experimental conditions are classified into 6 different clusters based on the expression profiles of two genes (*lexA* and *recA*). Cn represents the index of the cluster.

The regulatory interactions between TFs and their target genes vary under different experimental conditions [49]. DBoMM not only estimates the dependency of two genes, it can also identify the experimental conditions under which the predicted dependency occurs. In the reference regulatory network, it is known that *lexA* regulates the transcription of *recA* in SOS response [45], [46]. From Figure 4, DBoMM classifies the experiments into 6 clusters based on gene expression profile. For the first cluster, the expression level of *lexA* and *recA* are both low (8.7 and 8.5 respectively). When examining the samples in this cluster, we found 2 type of experiments: one is *recA* knock-out, and the other is addition of glucose and MgSO4 in the medium at the late log phase. We reasoned that when glucose is added into the media at the late log phase, the DNA replication and bacteria growth resume and the expression level of *lexA* and *recA* are low. We also found that cluster 4 and 5 (high expression of *lexA* and *recA*) mostly contain gene over-expression experiments, indicating that over-expression of these genes may activate *lexA*, which then up-regulate the *recA* expression. Compared to cluster 4 and 5, *recA* gene in cluster 6 is highly expressed whereas the expression of *lexA* are similar. Further examination revealed that cluster 6 includes two experiments: *recA* over-expression and norfloxacin treatment. This observation suggests that norfloxacin may activate the expression of *recA* but not *lexA*. Indeed, through literature search, we found that norfloxacin can inhibits DNA synthesis and cause an accumulation of single-stranded DNA fragments capable of activating the *RecA* protein [50]–[52].

These results demonstrate that DBoMM can provide important hints about the possible links among experimental conditions by clustering the similar experiments together. This feature can be very useful because it can guide experimental design for biologist to test the function of unknown genes.

## Discussion

In this paper, we describe a model-based method for gene dependency measurement based on gene expression profiles. As proposed by Segal [49], gene interactions may show similar or same pattern under different conditions. Based on this notion, we fit the gene expression profiles into a mixture Gaussian model. The experimental conditions are assigned into different components based on the similarity of regulatory interaction patterns. The difference between the joint and marginal distributions of gene expression profiles can then be used to describe the distance of two genes. We used the difference in BIC between the joint and the marginal distributions to estimate the overall dependency of genes. If the model is a simple component Gaussian distribution, which is equivalent to say the model is a regression model, 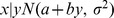, then our model is indeed purely based on the correlation. Our method extended the approaches using correlation because the advantage of the mixture model over correlation is: one simple correlation may not be able to describe the complex transcription process, and yet DBoMM can catch the different expression patterns under various experimental conditions. And the gene expression patterns reflect the conditional dependent regulatory interactions. Another advantage of the mixture model lies in its flexibility in choosing the component distributions. For example, we can use an additional Poisson distribution to handle the outliers in the dataset.

We have successfully validated the efficiency of DBoMM using *E.coli, Yeast, Drosophila, Arabidopsis* and synthetic datasets, and the results demonstrated that in general DBoMM performs the best compared to MI, COR, TAU and EUC. Specifically, DBoMM out-performed the other 4 methods using *Yeast, Arabidopsis* and synthetic dataset, and yet its performance is comparable to MI and COR respectively using the *E.coli* and *Drosophila* datasets. DBoMM does not require the linear relationships between genes and can catch both the local and the global correlations. Compared to the method calculating MI from expression profiles, DBoMM uses mixture model to estimate the probability, and can infer the experimental conditions under which the predicted regulatory interaction occurs.

In the 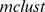 software, the mixture Gaussian model allows 10 covariance structures for multivariate cases and 2 in univariate cases [53], [54]. These covariance structures define the volume, shape and orientation of the distributions. Because of the complexity of the transcription process and experimental conditions, we chose the more general “VVV” model, (which allows volume, shape and orientation of distributions to be variable), to fit the gene expression profiles. For future work, we will further explore how the shape of the distribution may affect the model performance. In fact, DBoMM and MI adopt the similar strategy in the sense that they calculate the difference of variables based on the distribution difference. MI measures the mutual dependence of two random variables by using the difference between joint and marginal entropies. While DBoMM calculates the difference between joint and marginal mixture model distributions and takes into consideration of the model dimension. Detailed investigation of the theoretical as well as empirical relationships between DBoMM and MI can be an interesting future research topic.

We would also like to emphasize that DBoMM is only introduced as a new dependency measure instead of a complete network inference method. It means that DBoMM can be combined with many machine learning or existing network reconstructing methods to infer networks. For example, the dependency matrix composed of pairwise DBoMM values can also be used for gene clustering by employing a hierarchical clustering algorithm.

## Materials and Methods

### Data Sets

In this work, 4 compendiums of gene expression data including *E.coli, Yeast, Drosophila*, and *Arabidopsis* are used. Because the real regulatory interactions are far from completion, we use the synthetic dataset for method evaluation.

The *E.coli* gene expression data consist of 445 Affymetrix Antisense2 measuring the expression profiles (http://m3d.bu.edu/) of 4345 genes [55]. The microarrays were collected under different experimental conditions, such as PH changes, growth phases, antibiotics, heat shock, different media, varying oxygen concentrations and numerous genetic perturbations. RMA was used to normalize the data [56].

The regulation data is extracted from RegulonDB version 7 [39]. Of all the interactions, we removed these genes that do not match the probe sets and self-regulation interactions, leaving a reference network with 1531 non-redundant genes and 3774 experimentally confirmed regulatory interactions.

For *Yeast*, data package “yeastCC” [57] that includes a compendium of 77 cell cycle microarray expression profiles for 6178 genes [58] was used. We use “impute” package [59] to impute the missing expression data.

The *Yeast* gene interactions are extracted from YEASTRACT database [40]–[42], a curated repository with more than 48333 regulatory associations between transcription factors (TF) and target genes, based on more than 1200 bibliographic references. We removed the genes that do not match the probe sets and self-regulation interactions, leaving a reference network with 5898 non-redundant genes and 46000 regulatory interactions.

We also extract a compendium of 102 microarray expression profiles for early *Drosophila* development using 18952 probes [60], [61].

The *Drosophila* gene interactions are derived from DroID database [62], [63]. We removed the genes that do not match the probe sets and self-regulation interactions, leaving a reference network of 11509 non-redundant genes and 136522 regulatory interactions.

For *Arabidopsis*, 202 Affymetrix microarray measuring 22810 probes under 8 abiotic stress conditions, i.e. cold, osmotic, salt, drought, genotoxic, UV-B, wounding and heat [64], [65] treated are used.

The *Arabidopsis* gene interaction data are extracted from AGRIS database [66], [67]. We removed the genes that do not match the probe sets and self-regulation interactions, leaving a reference network of 6801 non-redundant genes and 9199 regulatory interactions.

We use SynTReN [48] to generate a simulated data set with various numbers of conditions and form a synthetic transcription regulatory network containing 1000 genes (Figure S4).

SynTReN is used to generate 5 simulated data sets with 100 experimental conditions and 500 genes for robustness estimation. Different level (0%, 20%,40%,60% and 80%) of biological and experimental noise is introduced to the simulated data.

### Dependency Measures

The Euclidean distance, Pearson correlation, Mutual information (MI),and Kendall’s tau correlation are commonly used measures in gene expression analysis. These methods quantify a pairwise distance or similarity between expression profiles over 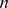 conditions that are represented by the two vectors 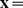 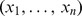, and 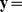 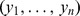.

#### Euclidean Distance, Pearson Correlation and Kendall’s tau correlation

The Euclidean distance between two expression profiles is given by
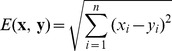
The Pearson correlation coefficient between two expression patterns is defined as



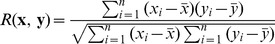
where 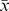, 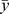 denote the average patterns level.

The Kendall’s 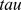 correlation between two expression patterns is:
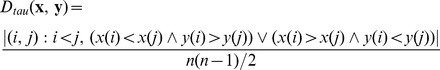



We used commands 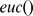, 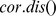 and 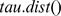 in package 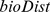 [68] under 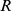 platform [69], [70] to calculate the Euclidean distance, Pearson correlation coefficient and Kendall’s tau correlation.

#### Mutual information

Given two random variables 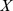, 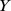 with respective ranges 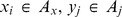 and probability mass functions 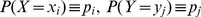, the Mutual information between two expression patterns, represented by random variables 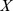 and 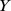, is given by
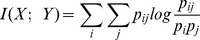



The gene expression profiles are divided into different bins and then the mutual information is computed. The data is treated as if they are discrete. We used 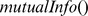 in package 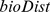 [68] and the default number of bins (10) to calculate the mutual information of two genes.

### Bayesian Information Criterion (BIC)

In statistics, the Bayesian information criterion (BIC) [71] is a criterion for model selection among a class of parametric models with different numbers of parameters. The formula for the BIC is described as:
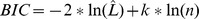
where 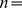
the number of data points, the number of observations, or equivalently, the sample size;


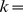
the number of free parameters to be estimated;


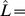
the maximized value of the likelihood function for the estimated model.

### Difference in BIC of Mixture Model (DBoMM)

The likelihood ratio between the joint distribution model and the independent marginal distribution models is often used to test the independency between two genes. Here, we use mixture Gaussian distributions to model gene expression profiles, because the mixture distribution can capture conditional dependent interactions between genes [37], [38].

To fit the expression profile of genes into the mixture model with the best number of components, we use Expectation-Maximization algorithms (EM) [72] to optimize the likelihood. We then use Bayesian Information Criterion (BIC) [71] to quantify the fitness of the model to the data and choose the number of mixture components. More details of the inference process can be found in Figure S5. Then the log-likelihood ratio
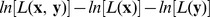
where 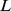 is the likelihood function given the model, can be calculated to test the independence of the two gene profiles 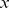 and 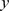.

In model selection literature [71], it is well known that the dimension of the model shall be penalized when searching for the best model. Therefore it is more preferable to compare the model probability instead of the likelihood in order to measure the gene dependency. This motivated the modification of the log-likelihood ratio to the difference of BIC between joint and marginal distribution models, which is defined as:
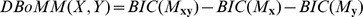
where 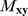 is the joint distribution model with minimal BIC of genes 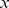 and 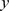, 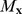 and 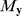 are marginal distribution models with minimal BIC of gene 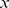 and gene 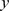 respectively. It turns out that DBoMM performs better than that of likelihood in most cases (Figure S6) when used for detecting the dependency of two genes’ expression profiles.


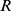
 [69], [70] package 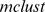 [53], [54] was used to fit the gene expression profiles into a mixture Gaussian distribution. And 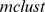 choose the number of components in a mixture model by the value that optimizes the BIC. In fact, 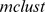 allows 10 different covariance structures for multivariate and 2 for univariate [54]. Because the transcription process is very complex and we know little prior knowledge about the joint expression profiles of genes under different conditions, we used the “VVV” model to describe the joint distribution of genes, which means the volume, shape and orientation of the covariance are variable.

### DBoMM can Distinguish Real Gene Interactions from the Background

**Figure 5:**
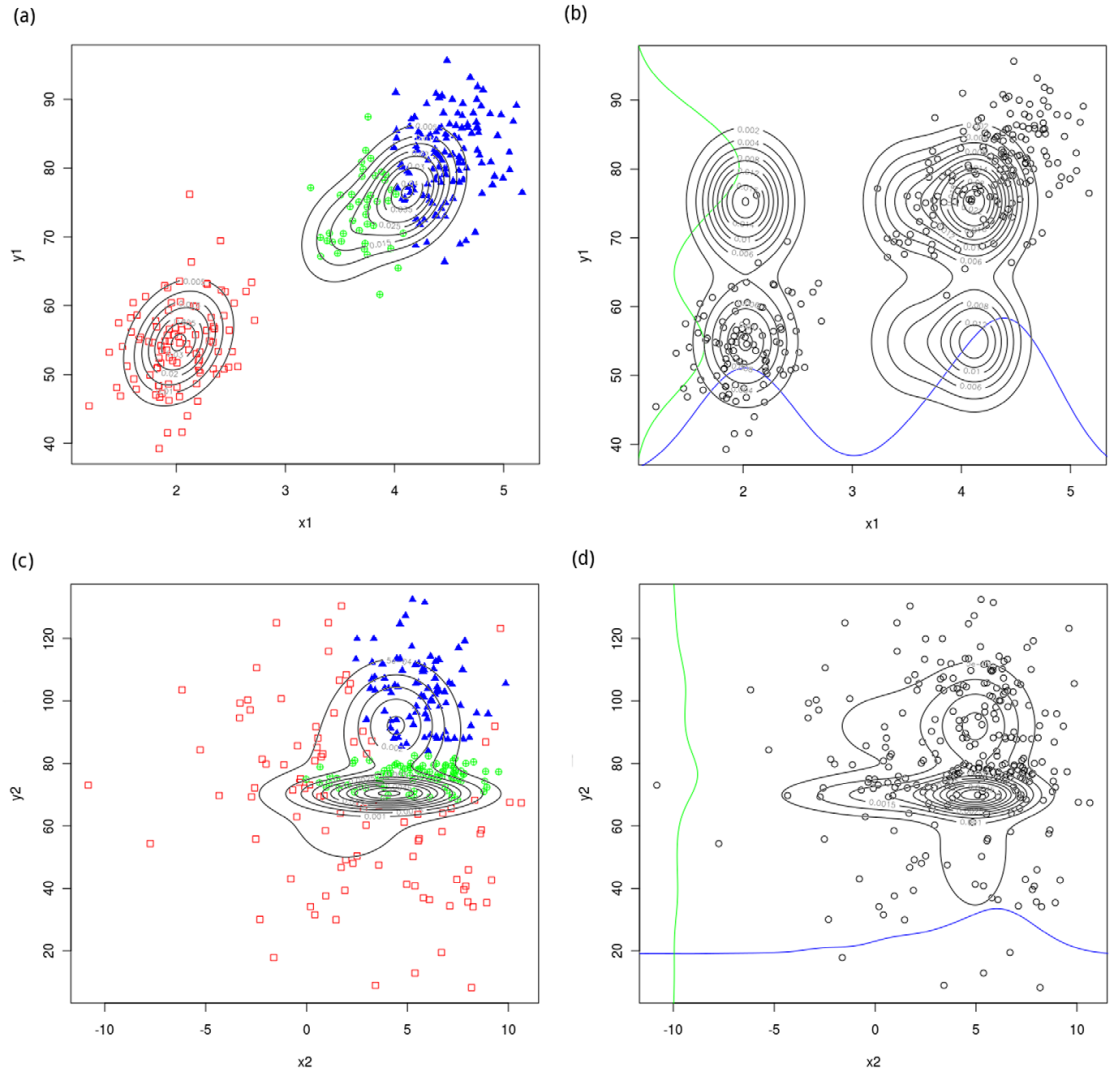
DBoMM can catch the conditional dependent interactions and distinguish the real gene interactions from the background. The expression profiles of two interacting genes (a) and non-interacting genes (c) are fitted into a bivariate mixture Gaussian distribution (joint distribution with different colors). The expression profiles of two interacting genes (b) and non-interacting genes (d) are separately fitted into two univariate mixture Gaussian distribution (marginal distribution). The blue and green lines represent the distribution of the two genes respectively. The contours correspond to the joint densities implied by DBoMM.

In order to examine the ability of DBoMM in distinguishing real gene interactions from the background, we first generate a synthetic gene expression dataset including 2 interacting gene x1 and y1 (Figure 5a,b) and 2 non-interacting gene x2 and y2 (Figure 5c,d). As shown in Figure1, the DBoMM model catches the local characters (different distributions) of the expression profiles and elucidates the conditional dependence of genes x1 and y1. Although the expression profiles of genes x2 and y2 also fit into 3 different distributions, the probability values of the expression profiles in joint distribution are low (because of the overlapping of the distributions and more scattered points in one distribution), indicating the weak or non dependence (global or local) between the two genes. The contours of the joint density implied by DBoMM are clearly different in the interaction case, while quite similar in the non-interaction case. This clearly demonstrated the discriminative ability of DBoMM.

### Measure the Performance of Different Methods

To compare the performance of different dependency measures, we computed the precision and recall of inferred networks by comparing the inferred networks to the reference network. Specifically, we produced one inferred networks for one giving pruning thresholds. Only interactions with scores above the pruning threshold were reported as links in the inferred network. Precision is the fraction of predicted interactions that are correct, i.e., TP/(TP + FP), and recall is the fraction of all known interactions that are discovered by the algorithm, i.e., TP/(TP + FN), where TP is the number of true positives, FP is the number of false positives, and FN is the number of false negatives. Precision and recall are computed over a range of pruning thresholds to produce the PR-curve. We constrained the resulting network maps to include only the genes available in the control set.

In practice, one threshold shall be selected for DBoMM in order to report one inferred network. By referring to the connection between BIC and posterior model probabilities, zero is one natural choice as the threshold of DBoMM. However, if there is training data available, the threshold of DBoMM can be set easily based on required precision or recall.

## Supporting Information

Figure S1 **A comparison of different methods using PR-curve based on the synthetic dataset.** X axis: recall; Y axis: precision. DBoMM out-performs other 4 methods using synthetic dataset.
(PNG)

Figure S2 **The recovered regulation network with 60% precision using** ***E.coli*** **dataset.** Pink and blue circles correspond to the transcription factors and target genes respectively. The size of the circle corresponds to the out-degree of gene in this network. Green arrows represent the interactions including in RegulonDB.
(ZIP)

Figure S3
**Motifs detected for transcription factor 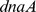 and 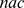.** (a).The 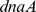 regulatory motif detected in the promoters of the 6 inferred target operons(upper) compared to the motif identified in PRODORIC(lower). (b). The 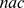 regulatory motif detected in the promoters of 11 inferred target operons(upper) compared to the motif identified in PRODORIC(lower).(PDF)

Figure S4
**Performances of 4 methods under various noise datasets.** (a). Mutual information(MI); (b). Pearson correlation(COR); (c). Euclidean distance(EUC); (d). Kendall’s 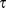 correlation(TAU).(PNG)

Figure S5 **The mixture model and algorithm of EM.** The multivariate Gaussian mixture model and the parameters estimation by using Expected Maximization algorithm.
(PDF)

Figure S6 **Performances of 6 methods(including the difference of likelihood) under various datasets.** (a). *E.coli* dataset; (b). *Yeast* dataset; (c). *Arabidopsis* dataset; (d). *Drosophila* dataset; In most cases, the difference of BIC between joint and marginal distribution models performs better than that of likelihood.
(PNG)
