# Supplementary figures and images for: A Model-Based Method for Gene Dependency Measurement
Source: PLoS One. 2012 Jul 19;7(7):e40918. doi: 10.1371/journal.pone.0040918 (PMC3400631; doi:10.1371/journal.pone.0040918)

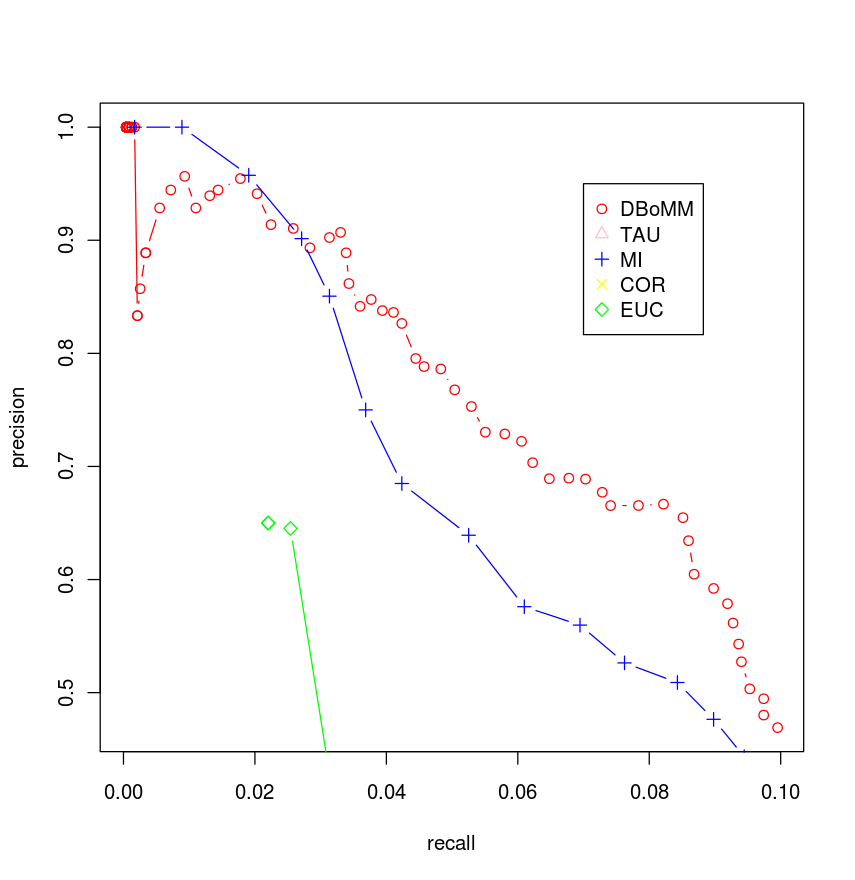

Supplement: Figure S1 — A comparison of different methods using PR-curve based on the synthetic dataset. X axis: recall; Y axis: precision. DBoMM out-performs other 4 methods using synthetic dataset. (PNG) [file pone.0040918.s001.png]

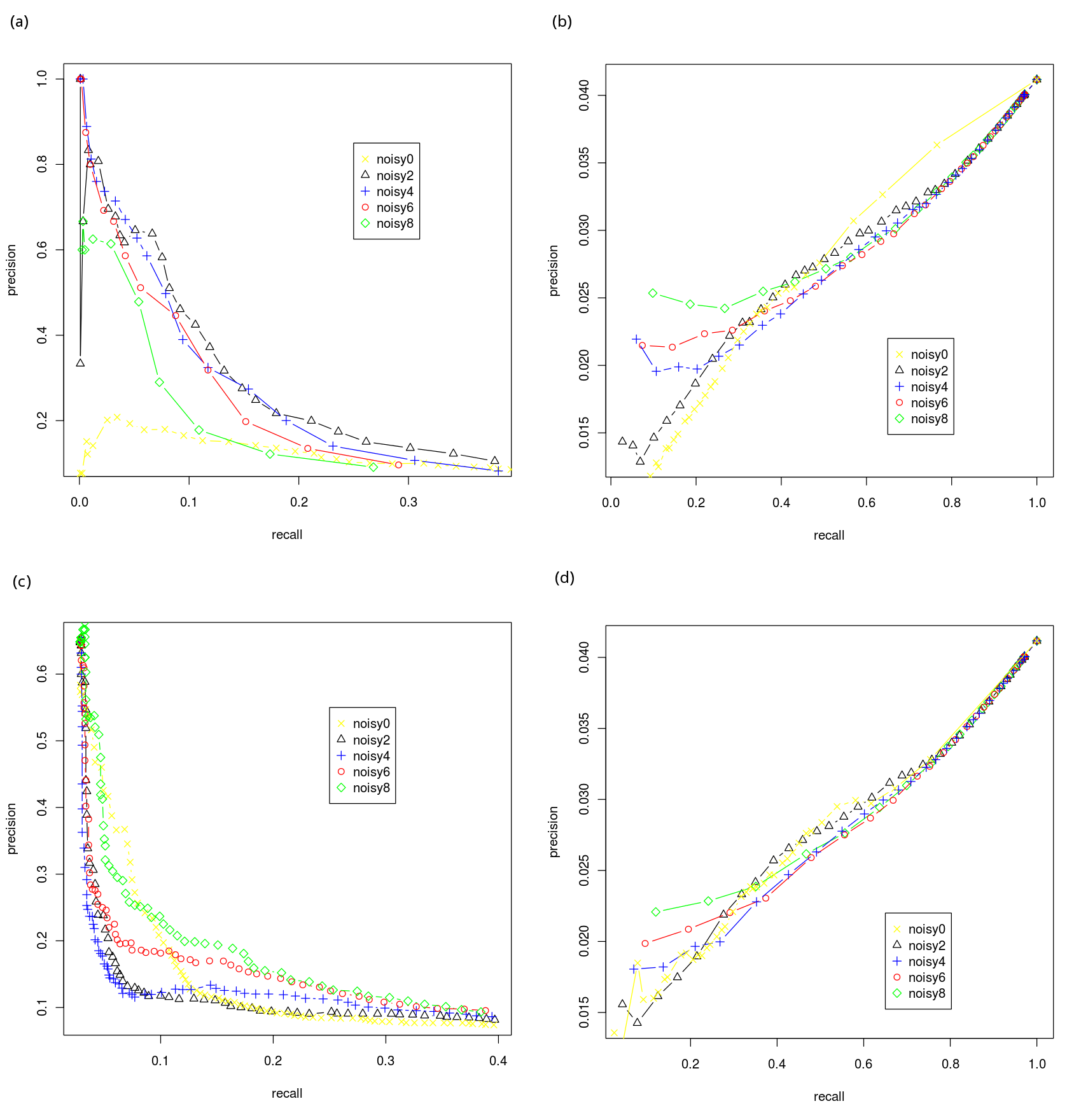

Supplement: Figure S4 — Performances of 4 methods under various noise datasets. (a). Mutual information(MI); (b). Pearson correlation(COR); (c). Euclidean distance(EUC); (d). Kendall’s correlation(TAU). (PNG) [file pone.0040918.s004.png]

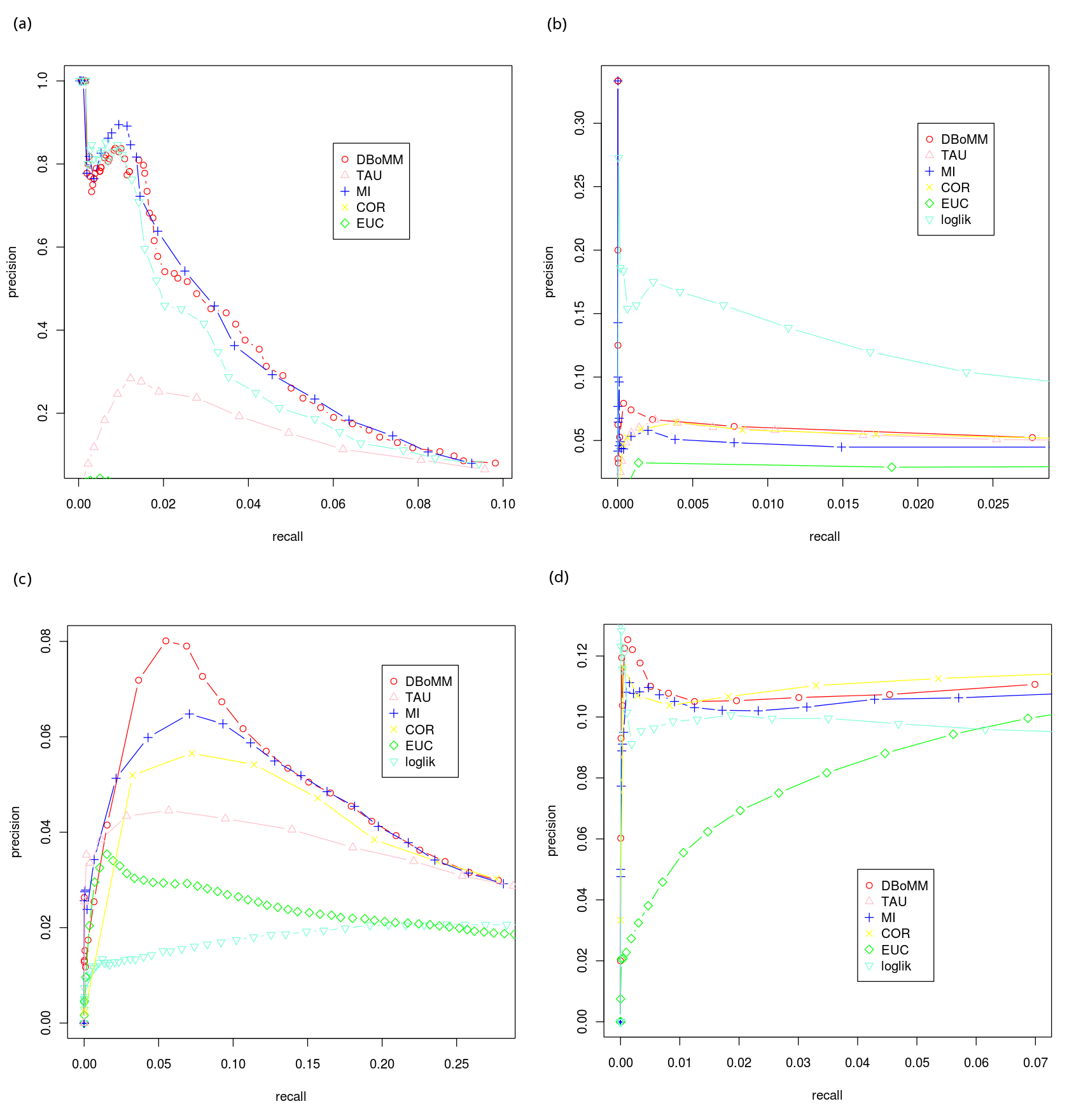

Supplement: Figure S6 — Performances of 6 methods(including the difference of likelihood) under various datasets. (a). E.coli dataset; (b). Yeast dataset; (c). Arabidopsis dataset; (d). Drosophila dataset; In most cases, the difference of BIC between joint and marginal distribution models performs better than that of likelihood. (PNG) [file pone.0040918.s006.png]
