# Supplementary material for: A Model-Based Method for Gene Dependency Measurement
Source: PLoS One. 2012 Jul 19;7(7):e40918. doi: 10.1371/journal.pone.0040918 (PMC3400631; doi:10.1371/journal.pone.0040918)
Supplement: Figure S2 — The recovered regulation network with 60% precision using E.coli dataset. Pink and blue circles correspond to the transcription factors and target genes respectively. The size of the circle corresponds to the out-degree of gene in this network. Green arrows represent the interactions including in RegulonDB. (ZIP) [file pone.0040918.s002.zip › S2/S2.pdf]

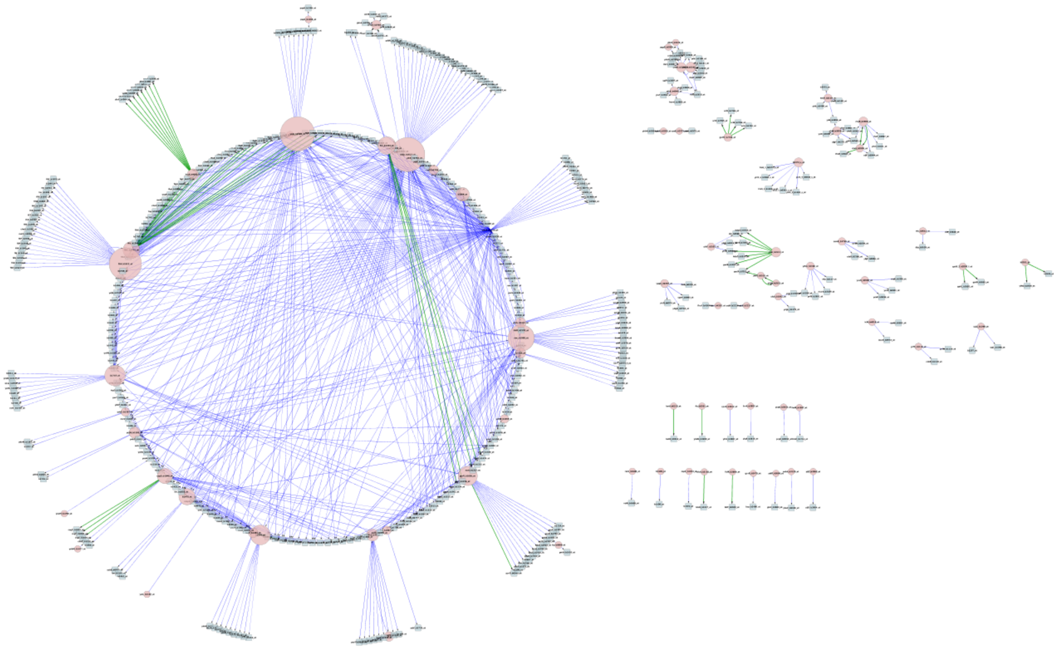

Figure S2. The recovered regulation network with 60% precision based on *E.coli* dataset. Pink and blue circles correspond to the transcription factors and target genes respectively. The size of the circle corresponds to the out-degree of gene in this network. Green arrows represent the interactions including in RegulonDB.
