# Supplementary material for: A Model-Based Method for Gene Dependency Measurement
Source: PLoS One. 2012 Jul 19;7(7):e40918. doi: 10.1371/journal.pone.0040918 (PMC3400631; doi:10.1371/journal.pone.0040918)
Supplement: Figure S3 — Motifs detected for transcription factor and . (a).The regulatory motif detected in the promoters of the 6 inferred target operons(upper) compared to the motif identified in PRODORIC(lower). (b). The regulatory motif detected in the promoters of 11 inferred target operons(upper) compared to the motif identified in PRODORIC(lower). (PDF) [file pone.0040918.s003.pdf]

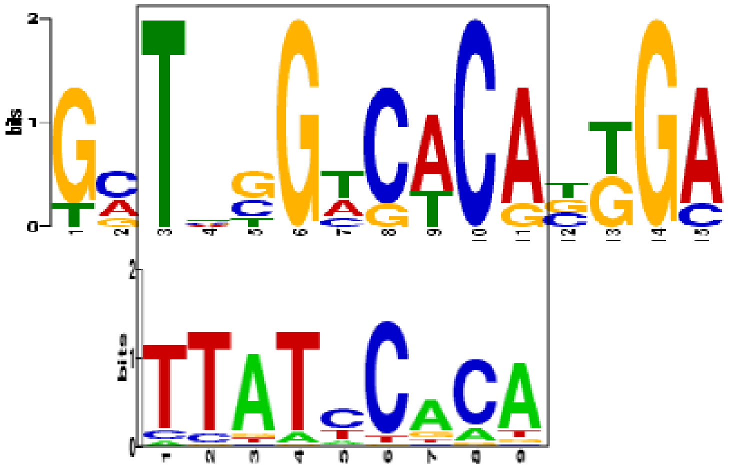

Figure S3 (a). Motif detected for transcription factor *dnaA*. The *dnaA* regulatory motif detected in the promoters of the 6 inferred target operons(upper) compared to the motif identified in PRODORIC(lower).

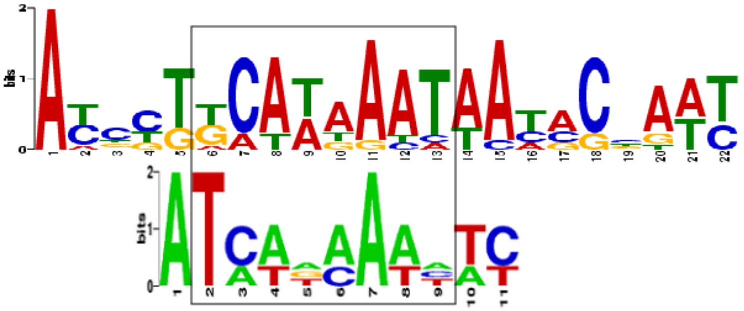

Figure S3 (b). Motif detected for transcription factor *nac*. The *nac* regulatory motif detected in the promoters of 11 inferred target operons(upper) compared to the motif identified in PRODORIC(lower).
