# Supplementary material for: A Model-Based Method for Gene Dependency Measurement
Source: PLoS One. 2012 Jul 19;7(7):e40918. doi: 10.1371/journal.pone.0040918 (PMC3400631; doi:10.1371/journal.pone.0040918)
Supplement: Figure S5 — The mixture model and algorithm of EM. The multivariate Gaussian mixture model and the parameters estimation by using Expected Maximization algorithm. (PDF) [file pone.0040918.s005.pdf]

Multivariate Gaussian mixture model

We use multivariate Gaussian mixture model to describe the expression of genes. The joint probability of gene expression value is

$$p(\mathrm{g}_1, \cdots, \mathrm{g}_D) = \sum_{k=1}^{K} \pi_k N(\boldsymbol{\mu}_k, \boldsymbol{\Sigma}_k)$$

where $\{\mathrm{g}_1, \cdots, \mathrm{g}_D\}$ are genes, π_k is the weight of the kth component and $\sum_{k=1}^{K} \pi_k = 1$, $\boldsymbol{\mu}_k$ and $\boldsymbol{\Sigma}_k$ are the mean vector and the covariance matrix of the kth component respectively .

Parameter estimation

Suppose we have a set of genes $\{\mathrm{g}_1, \cdots, \mathrm{g}_D\}$ in one module and there are N expression data points $\{\boldsymbol{e}_1, \ldots, \mathbf{e}_N\}$, this data set can be represented as an $N \times D$ matrix $\boldsymbol{E}$. We assume the N data points are independent from the same multivariate Gaussian mixture distribution. The log-likelihood of the observed data is :

$$\ln p(\boldsymbol{E}|\boldsymbol{\pi}, \boldsymbol{\mu}, \boldsymbol{\Sigma}) = \sum_{n=1}^{N} \ln \left\{ \sum_{k=1}^{K} \pi_k N(\boldsymbol{e}_n | \boldsymbol{u}_k, \boldsymbol{\Sigma}_k) \right\}$$

The training process is to find the maximum likelihood estimate of $(\pi, \mu, \sum)$. An elegant and powerful method for handling this task is the $Expectation - Maximization$ (EM) algorithms.

E step: Evaluate the responsibilities using the current parameter values

$$\gamma(z_{nk}) = \frac{\pi_k N(\boldsymbol{e}_n | \boldsymbol{\mu}_k, \boldsymbol{\Sigma}_k)}{\sum_{j=1}^{K} \pi_j N(\mathbf{e}_n | \boldsymbol{\mu}_j, \boldsymbol{\Sigma}_j)}$$

$\gamma(z_{nk})$is the posterior probability that component k is responsible for generating $\boldsymbol{e}_n$.

M step: Re-estimate the parameters using the current responsibilities

$$\boldsymbol{\mu}_k^{\mathrm{new}} = \frac{1}{N_k} \sum_{n=1}^{N} \gamma(z_{nk}) \boldsymbol{e}_n$$

$$\boldsymbol{\Sigma}_k^{\mathrm{new}} = \frac{1}{N_k} \sum_{n=1}^{N} \gamma(z_{nk}) (\boldsymbol{e}_n - \boldsymbol{\mu}_k^{\mathrm{new}})(\boldsymbol{e}_n - \boldsymbol{\mu}_k^{\mathrm{new}})^{\mathrm{T}}$$

$$\pi_k^{\mathrm{new}} = \frac{N_k}{N}$$

where

$$N_k = \sum_{n=1}^{N} \gamma(z_{nk})$$
